# Apple latent spherical virus (ALSV)-induced gene silencing in a medicinal plant, *Lithospermum erythrorhizon*

**DOI:** 10.1038/s41598-020-70469-1

**Published:** 2020-08-11

**Authors:** Yuki Izuishi, Natsumi Isaka, Hao Li, Kohei Nakanishi, Joji Kageyama, Kazuya Ishikawa, Tomoo Shimada, Chikara Masuta, Nobuyuki Yoshikawa, Hiroaki Kusano, Kazufumi Yazaki

**Affiliations:** 1 Laboratory of Plant Gene Expression, Research Institute for Sustainable Humanosphere, Kyoto University, Gokasho, Uji 611-0011 Japan; 2 Graduate School of Science, Kyoto University, Sakyo-ku, Kyoto, 606-8502 Japan; 3 Research Faculty of Agriculture, Hokkaido University, Kita 9 Nishi 9, Kita-ku, Sapporo, 060-8589 Japan; 4 Agri-Innovation Center, Iwate University, Morioka 3-18-8, Iwate, 020-8550 Japan

**Keywords:** Biological techniques, Biotechnology, Molecular biology, Plant sciences

## Abstract

*Lithospermum erythrorhizon* is a medicinal plant that produces shikonin, a red lipophilic naphthoquinone derivative that accumulates exclusively in roots. The biosynthetic steps required to complete the naphthalene ring of shikonin and its mechanism of secretion remain unclear. Multiple omics studies identified several candidate genes involved in shikonin production. The functions of these genes can be evaluated using virus-induced gene silencing (VIGS) systems, which have been shown advantageous in introducing iRNA genes into non-model plants. This study describes the development of a VIGS system using an apple latent spherical virus (ALSV) vector and a target gene, phytoene desaturase (*LePDS1*). Virus particles packaged in *Nicotiana benthamiana* were inoculated into *L. erythrorhizon* seedlings, yielding new leaves with albino phenotype but without disease symptoms. The levels of *LePDS1* mRNAs were significantly lower in the albino plants than in mock control or escape plants. Virus-derived mRNA was detected in infected plants but not in escape and mock plants. Quantitative PCR and deep sequencing analysis indicated that transcription of another hypothetical *PDS* gene (*LePDS2*) also decreased in the defective leaves. Virus infection, however, had no effect on shikonin production. These results suggest that virus-based genetic transformation and the VIGS system silence target genes in soil-grown *L. erythrorhizon*.

## Introduction

*Lithospermum erythrorhizon* is a Boraginaceaeous medicinal plant that produces a unique red naphthoquinone, shikonin, which accumulates exclusively in its roots. The dried roots have been used as a crude drug in Asian countries, with shikonin derivatives being major active pharmaceutical components of these herbal medicines. Shikonin and its stereo-isomer alkannin have been reported to have various biological activities, including antibacterial, anti-inflammatory, anti-oxidant, antitumor, anti-angiogenic^1^, and anti-topoisomerase^2^ activities. These compounds have also been found to enhance granulation and glucose uptake^3^ and to reduce adiposity^4^. These natural pigments are also used as dyes, especially for cloth, and to have other commercial uses. Although shikonin appears to be a simple compound, many steps are necessary for its chemical synthesis, making the supply of shikonin exclusively dependent on natural resources. *L. erythrorhizon*, however, is an endangered species, preventing widespread continuous cultivation.

Biologic approaches are currently used to synthesize high value plant products in unicellular organisms, such as yeasts. Production of a target compound, however, requires the determination of its entire biosynthetic pathway and the identification of all genes and proteins involved in its biosynthesis. Shikonin is a meroterpene synthesized via two independent biosynthetic pathways, a common phenylpropanoid pathway and an isopreniod pathway^5^. Although several enzymes and genes involved in the initial steps of shikonin biosynthesis have been identified to date^6–10^, genes and proteins involved in later steps have not yet been identified, especially those involved in the crucial steps required to form the naphthalene ring.

To identify candidate genes involved in these later steps of shikonin biosynthesis, we performed large-scale transcriptome and comparative proteome analyses, based on results showing that shikonin production can be strictly regulated by many chemical and physical factors^9^. For example, shikonin production is strongly inhibited by ammonium ion and illumination, but is markedly enhanced in M9 medium. Shikonin production is also tissue specific, being produced solely in the epidermis, but not in other root tissues, such as inside the central cylinder^6^. These characteristics enabled the identification of more than 10 putative candidate genes involved in shikonin biosynthesis^9^ and more than 16 putative candidate genes involved in shikonin secretion from cells^11^.

These genes of interest may be characterized using the hairy root transformation technique developed in *L. erythrorhizon*. This method can be used to introduce an exogenous gene for overexpression or an RNAi to suppress the expression of an endogenous gene^12^. The recent development of a new protocol has dramatically improved the transformation efficiency of this system^13^. However, stable transformation is time-consuming, with several months required to evaluate the physiological function of a gene. The present study utilized a virus-induced gene silencing (VIGS) system to establish a method for functional analyses of genes of interest in *L. erythrorhizon* plants.

One major advantage of VIGS is that special equipment is not required to introduce a foreign gene fragment into a non-model plant. Within a short period of time after infection, the virus becomes wide-spread throughout the entire plant body, allowing evaluation shortly after infection. One disadvantage of VIGS is that the host-virus interaction is highly species-specific. Thus if a plant virus is unable to infect a plant species, the introduced gene fragment cannot be evaluated. This study assessed the abilities of apple latent spherical virus (ALSV) and cucumber mosaic virus (CMV), both domestic plant viruses found in Japan, to infect *L. erythrorhizon*.

## Results

ALSV has been shown to infect a broad spectrum of plant species, and has been used as a viral vector including for gene silencing^14^. To determine if ALSV can infect *L. erythrorhizon*, we assessed its ability to knock down expression of the phytoene desaturase (*PDS*) gene in this plant species. Successful infection and *PDS* gene silencing should result in the appearance of albino leaves, a representative phenotype of the knockdown of *PDS* function^15,16^.

### ALSV-mediated VIGS phenotype of the *PDS* gene in *L. erythrorhizon*

**Figure 1:**
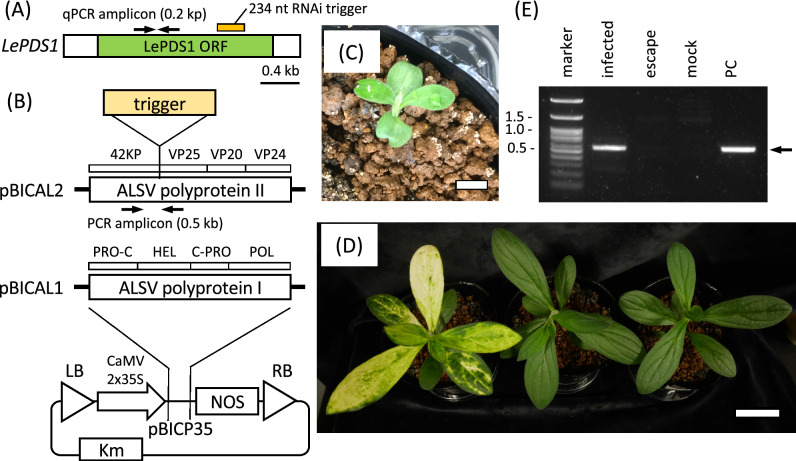
Generation of albino leaves in *L. erythrorhizon* plants by infection with ALSV containing a VIGS trigger against *LePDS1*. (**A**) Schematic representation of the *LePDS1* transcript. The VIGS trigger fragment and PCR primers were set on the region encoding *LePDS1* protein. (**B**) Construction of an ALSV vector. The trigger fragment was inserted in the reading frame of the ALSV polyprotein without any stop codons or frame shifts. The PCR primers to detect ALSV included the trigger fragment within the amplicon, with the product expected to be 0.5-kb long. The bipartite ALSV RNAs were encoded downstream of the CaMV 2 × 35S promoter in the pBICP35-based plasmids (pBICAL2 and pBICAL1). Abbreviations: LB and RB, T-DNA borders; 42 KP, 42 K movement protein; VP25, VP20 and VP24, capsid proteins; PRO-C, protease cofactor; HEL, NTP-binding helicase; C-PRO, cysteine protease; POL, RNA polymerase; NOS, terminator. (**C**) Young seedlings of *L. erythrorhizon* just before inoculation. Scale bar = 1 cm. (**D**) *L. erythrorhizon* plant grown for five weeks after inoculation. Left, an "infected" plant with albino leaves; center, an "escape" plant without albino leaves despite virus inoculation; right, a “mock” infected plant. Scale bar = 3 cm. (**E**) Detection of ALSV in *L. erythrorhizon* by RT-PCR. Abbreviations: infected, defective plant with albino leaves; escape, healthy plant despite virus inoculation; mock, plant treated with inoculation solution without virus; PC, positive control of PCR. The arrow indicates the band size corresponding to the PCR amplified fragment.

Transcriptome analysis of *L. erythrorhizon* identified a *PDS* gene, designated *LePDS1* (accession no. LC512725). A 234 nucleotide fragment of this gene (Fig. 1A) was cloned into ALSV using a method involving rub-inoculation following *Agribactrium*-mediated inoculation of *Nicotiana benthamiana* (see the Methods section for more detail) (Fig. 1B)^17^. The packaged virus was inoculated onto the leaves of young *L. erythrorhizon* seedlings during the stage of first leaf expansion (Fig. 1C). After cultivation for about 3 weeks, newly developed leaves above the inoculated leaves of several plants showed albino phenotypes. Although the albino phenotype was reproducibly observed, about 70% of inoculated seedlings did not have albino leaves and were considered to have escaped infection (Fig. 1D). Viral RNA was detected in infected plants that showed the albino phenotype, but not in plants that escaped infection or in mock control plants, which were treated with water instead of ALSV (Fig. 1E).

### Abundance of *PDS* transcript in infected *L. erythrorhizon*

**Figure 2:**
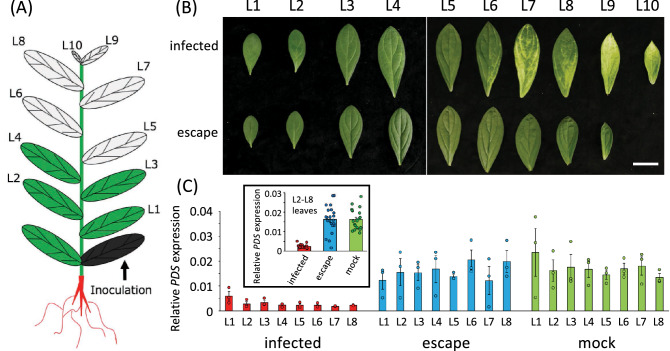
Abundance of *LePDS* mRNA in ALSV infected plants. (**A**) Schematic representation of an *L. erythrorhizon* plant with expansion of 12 true leaves. One of the first four leaves of the seedlings, including the cotyledon, was inoculated with virus. L1–L10 represent true leaves 3–12, respectively. (**B**) Leaves from infected and escape plants. Scale bar = 3 cm. (**C**) Relative *LePDS* mRNA expression measured by qRT-PCR normalized relative to actin 7 (*LeACT7*) mRNA expression in the same samples. Error bars represent standard errors (n = 3). The box indicates comparisons of leaves L2–L8 in infected, escape, and mock infected plants (n = 21).

The expression of *PDS* mRNA was assessed by qRT-PCR five weeks after viral inoculation of *L. erythrorhizon* leaves located at levels ranging from L1 (older) to L10 (younger) toward the shoot tips (Fig. 2A). The albino phenotype was observed in leaves at L5–L10, but not in leaves at L1–L4 (Fig. 2B). To compare the expression of *PDS* mRNA detected by qRT-PCR in leaves and plants, its level was normalized to that of an internal standard, actin 7 (*LeACT7*) mRNA (Fig. 2C), which is ubiquitously expressed in these plants^10^. *PDS* mRNA expression was lower in infected than in escape or mock plants, with the differences in *PDS* mRNA expression at L2–L8 being significantly lower in infected than in escape (p = 4.08 × 10^–11^) or mock infected (p = 6.89 × 10^–15^) plants, with no difference between escape and mock infected plants (p = 0.53). A comparison of the relative expression of *PDS* mRNA in each leaf of each infected, escape, and mock control plant showed that *PDS* expression was almost constitutive, with differences within plants being generally statistically not significant, although *PDS* expression was significantly higher in L1 than in other leaves of infected plants (p = 0.014). These results strongly suggest that *PDS* expression was significantly inhibited by ALSV-mediated VIGS in infected plant leaves.

### Effect of virus infection on shikonin biosynthesis in roots

**Figure 3:**
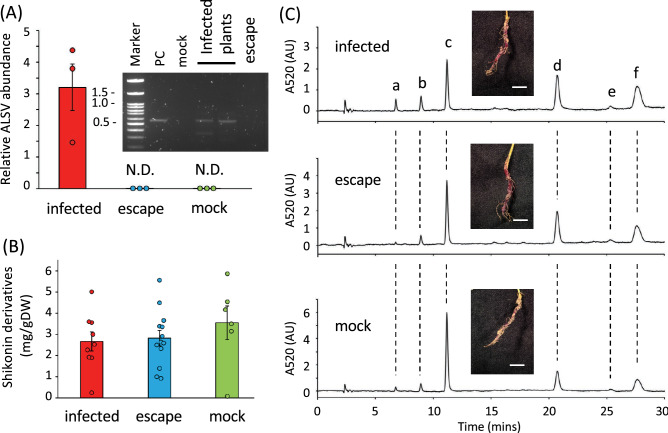
Biosynthesis of shikonin derivatives in ALSV infected *L. erythrorhizon* roots. (**A**) Relative abundance of ALSV RNA in roots, as measured by qRT-PCR and normalized relative to actin 7 (*LeACT7*) mRNA expression. Also shown is a gel image of the PCR products. Error bars represent standard errors (n = 3). ND, no amplicon detected by qPCR. (**B**) Spectrophotometric measurements of amounts of shikonin derivatives in root extracts. Error bars represent standard errors (infected, n = 9; escape, n = 13; mock, n = 6). (**C**) Representative photographs of roots and HPLC chromatograms of the extracts. The compounds were identified by comparison with standards. a, shikonin; b, α-hydroxyisovalerylshikonin; c, acetylshikonin; d, isobutyrylshikonin; e, β,β-dimethylacrylshikonin; f, β-methylbutylshikonin.

Because VIGS in *L. erythrorhzon* was developed to suppress genes of interest involved in shikonin production, virus infection itself should not affect the production of shikonin. To assess whether ALSV infection altered shikonin biosynthesis, roots were harvested from virus- and mock-infected plants and shikonin derivatives were quantitatively analyzed (Fig. 3). Assessment of ALSV abundance in RNA samples from roots showed that, similar to leaves, the virus was present only in the roots of infected plants, not in the roots of escape and mock infected plants (Fig. 3A). HPLC analysis of the amounts and compositions of shikonin derivatives in the harvested roots showed no significant differences among the three plant types, indicating that ALSV infection of *L. erythrorhizon* plants did not influence their production of shikonin (Fig. 3B). The major shikonin derivatives in all three plant types were acetylshikonin, isobutyrylshikonin, and α-methylbutylshikonin, accompanied by minor derivatives, including shikonin, β-hydroxyisovalerylshikonin, and β,β-dimethylacrylshikonin (Fig. 3C). These results indicate that the shikonin biosynthesis pathway was not disturbed by ALSV infection of and proliferation in plant tissues, including in their roots, or by the silencing of a gene unrelated to shikonin production.

### Simultaneous ALSV silencing of *PDS* genes in *L. erythrorhizon*

**Figure 4:**
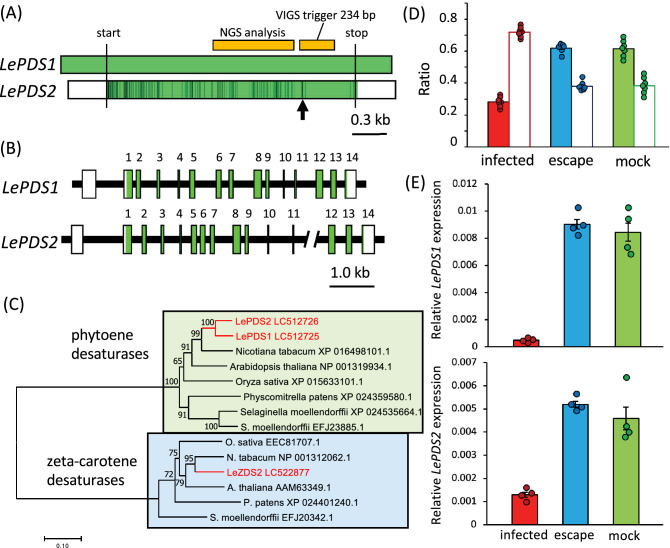
Simultaneous knockdown of PDS family genes by VIGS. (**A**) Schematic representation of *LePDS1* and *LePDS2* transcripts. The green color represents nucleotides identical in *LePDS2* and *LePDS1*; the black color represents mismatched nucleotides in *LePDS2* relative to *LePDS1*; and the white color represents regions in *LePDS2* non-homologous to those in *LePDS1*. The arrow indicates the mismatched region of the VIGS trigger relative to *LePDS2*. NGS analysis indicates the amplicon for deep sequencing. Scale bar = 0.3 kb. (**B**) Schematic representation of the genomic structures of *LePDS1* and *LePDS2*. Numbers represent coding exons, with coding regions indicated in green. Scale bar = 1.0 kb. (**C**) Neighbor-joining tree of the phytoene desaturase (green) and zeta-carotene desaturase (blue) protein families. The organism and the Genbank accession numbers for sequences are indicated. *L. erythrorhizon* proteins are labeled in red. Numbers represents bootstrap values of 1,000 replicates. Scale bar = substitution of 0.1 amino acid. (**D**) Read count ratio of *LePDS1* to *LePDS2* transcripts in leaves measured by deep sequencing. Filled bars represent *LePDS1*/total and white bars represent *LePDS2*/total. Error bars represent standard errors (n = 7). (**E**) Relative *LePDS1* and *LePDS2* expression calculated from the read count ratio and total *LePDS* expression measured by qRT-PCR normalized to actin 7 gene (*LeACT7*) expression in the same samples (n = 4).

Because our findings suggested that the albino phenotype was partial in *L. erythrorhizon*, we suspected that the silencing efficiency was not sufficient or that these plants also expressed an isoform of *PDS*. Careful search of our transcriptome data identified another *PDS* paralogue, *LePDS2* (accession no. LC512726), with a nucleotide sequence highly similar to that of *LePDS1*, including 91% identity in the coding region. *LePDS2* was found to encode a polypeptide of the same length as *LePDS1* (90% identity). The nucleotide sequences of the 5′UTR and 3′UTR regions of these two genes, however, differed markedly (Fig. 4A). Assembly of the genomic DNA sequence of *L. erythrorhizon* from the available Genbank dataset (SRP108575) showed that the *LePDS2* gene had the same exon–intron structure as 14 coding exons of *LePDS1,* but their lengths and intron sequences differed (Fig. 4B). A phylogenetic analysis of their amino acid sequences showed that LePDS1 and LePDS2 differed at 53 of 581 amino acid residues, but that these members of the phytoene desaturase family differed markedly from those of the zeta-carotene desaturase family (Fig. 4C). These findings suggested that LePDS1 and LePDS2 evolved in a recent ancestor of *L. erythrorhizon* after splitting from a single, broadly conserved *PDS* gene.

Compared with *LePDS1*, *LePDS2* differs at six nucleotides within the VIGS trigger (Fig. 4A). To evaluate the silencing efficiency of these two close paralogues by VIGS, the ratio of their transcript levels was evaluated by amplicon sequencing (Fig. 4D,E) using identical sequences of *LePDS1* and *LePDS2* (Fig. 4A). The read number of *LePDS2* was slightly but significantly smaller than that of *LePDS1* in escape (n = 7, p = 1.50 × 10^–9^) and mock-infected (p = 2.10 × 10^–6^) plant leaves, suggesting that the levels of expression of *LePDS1* and *LePDS2* are almost the same (6:4) in a normal environment (Fig. 4D). In infected plants, however, the read number of *LePDS1* was much smaller than that of *LePDS2* (n = 7, p = 8.19 × 10^–12^) (Fig. 4D) and the *LePDS1*:*LePDS2* ratio in infected (n = 7) plant leaves differed significantly from the ratios in escape (n = 14, p = 7.17 × 10^–11^) and mock infected (n = 14, p = 5.19 × 10^–9^) plant leaves (Fig. 4D). These results suggested that the designed sequence in this VIGS experiment preferentially knocked down *LePDS1* rather than *LePDS2,* although the expression of *LePDS2* was negatively regulated to a lesser extent. qRT-PCR measurements of individual and combined expression of *LePDS1* and *LePDS2* and of the *LePDS1*: *LePDS2* ratio in albino L5–L8 leaves expected by amplicon sequencing (Fig. 4E) showed that *LePDS1* expression was significantly lower in leaves of infected than of escape (n = 4, p = 1.17 × 10^–6^) and mock infected (n = 4, p = 7.00 × 10^–5^) plants, Similarly, *LePDS2* expression, was significantly lower in leaves of infected than of escape (n = 4, p = 1.22 × 10^–6^) and mock treated (n = 4, p = 1.68 × 10^–3^) plants. These results suggest that the VIGS simultaneously knocked down the expression of both the *LePDS1* and *LePDS2* genes. However, the silencing efficiency for both *LePDS* genes did not reach 100%, resulting in a pale green phenotype.

### Other plant virus vectors for VIGS in *L. erythrorhizon*

In Japan, the number of wild *L. erythrorhizon* plants has markedly decreased in the last several decades, with one of the major reasons being the susceptibility of these plants to plant viruses, such as cucumber mosaic virus (CMV). We therefore assessed the use of other virus vectors, such as CMV and tobacco rattle virus (TRV), for VIGS in *L. erythrorhizon*. Infection of these plants with domestic CMV yielded a dwarf phenotype (Fig. S1A,B), whereas infection with TRV did not induce a marked phenotypic change (Fig. S2). The use of TRV is strictly limited in Japan, as is the transfer of infectious materials from other countries. This limits further investigations with TRV, as this pathogenic virus originated in the United States.

A CMV vector for VIGS^18^ was used to silence the *L. erythrorhizon* dark-inducible gene-2 (*LeDI-2*), which is involved in shikonin production^19^, in cultured shoots, with a GFP fragment of nearly the same length used as a control (Fig. S1C,D). Similar to findings with ALSV, CMV silenced the *LeDI-2* gene in cultured shoots, parallel to the reduction in shikonin production (Fig. S1), suggesting that CMV can be utilized for VIGS in *L. erythrorhizon*.

## Discussion

The roots of *L. erythrorhizon* have been used in crude drugs and as natural dyes for many centuries. Because of its marked decrease in the wild, due both to overharvesting and the worldwide spread of plant viruses, *L. erythrorhizon* is considered an endangered species. Using a multiple omics approach, we have identified several genes that may be involved in shikonin biosynthesis and secretion. To analyze the function of each candidate gene and its possible involvement in shikonin biosynthesis and secretion, we established a VIGS system in *L. erythrorhizon*. Compared with the RNAi approach to analyzing gene function, in which hairy roots are generated using *Rhizobium rhizogenes*^13^, the VIGS approach is more rapid. Specifically, the RNAi-induced hairy root formation approach requires at least 6 weeks from infection to see the phenotype associated with suppression of the gene of interest. In contrast, the VIGS-mediated approach takes only about 3 weeks to observe these phenotypes.

ALSV is distributed thorough the body of various plant species. For example, ALSV was shown to be present in root tissues of soybean and pea plants, and ALSV-mediated VIGS was reported effective in these plant tissues^20,21^. This virus is ubiquitously distributed in most cell types, including the epidermis, of various plant species, making this virus useful for the knockdown of genes preferentially expressed in epidermal cells^22,23^. ALSV was able to infect *L. erythrorhizon* without inducing any obvious phenotypic differences. *PDS* was selected as a model gene for silencing of expression by VIGS. Knockdown of *PDS* in soil grown plants resulted in an albino phenotype without any growth defect. The albino appearance of newly developed leaves is a representative phenotype of the *PDS* gene in plant biology^15^. The spread of ALSV was also confirmed in the infected plant body, as the virus was also detected in root tissues in *L. erythrorhizon*. This finding is very important, as this study aimed to evaluate gene function in roots, the sole source of shikonin derivatives.

Shikonin production was not significantly affected by ALSV infection. Specifically, the total shikonin content and the composition of shikonin derivatives in the roots of infected plant roots were the same as those in the roots of both escape and mock infected plants. As shikonin derivatives specifically accumulate in the root epidermis of these plants, the wide distribution of ALSV, including in the epidermis, is also advantageous. Taken together, these findings suggest that ALSV-mediated VIGS may provide a powerful tool for analysis of the functions of genes involved in shikonin biosynthesis in *L. erythrorhizon* (Fig. S3).

Analysis of deep sequencing data from *L. erythrorhizon* identified three *PDS*-like genes, with two classified as being in the *PDS* gene family. These two genes may have diverged from a common *PDS* gene in a near ancestor of *L. erythrorhizon*, as we could not find two conserved *PDS* subfamilies among eudicots. Infection of plant leaves with virus containing the *LePDS1* construct resulted in simultaneous knockdown of both *PDS* genes, with *LePDS1* being more strongly suppressed. The VIGS trigger sequence contains two regions, 49 and 156 nucleotides in length, that match *LePDS2*, with these two regions separated by a 29 nucleotide-long sequence containing six nucleotide mismatches. In contrast, the full 234 nucleotide sequence of the VIGS trigger matches that of *LePDS1*. These findings suggest that this trigger sequence was effective in knocking down gene expression, but that knockdown of these two potentially functional *LePDS* genes showed statistically and physiologically significant differences.

A CMV-based vector also showed potential in gene silencing. CMV was found to infect *L. erythrorhizon*, inducing growth arrest insertion of a DNA fragment into the CMV vector usually decreased the disease phenotype, depending on insert length, with a longer insert associated with a weaker phenotype. We found that the CMV vector containing a fragment of the *LeDI-2* gene knocked down the expression of endogenous *LeDI-2* in the shoots of *L. erythrorhizon*, with the production of shikonin derivatives being slightly but not significantly reduced in these shoots. We previously reported that *LeDI-2* antisense RNA knocked down *LeDI-2* expression and the production of shikonin derivatives in hairy root cultures^19^. The results presented in this study were similar, suggesting that CMV may act to suppress targeted genes in *L. erythrorhizon*.

Shikonin derivatives are secreted by cells and accumulate in the apoplastic space. The molecular mechanism underlying shikonin secretion is of great importance. Shikonin is a very lipophilic metabolite and rapidly crystallizes under aqueous conditions. Plants contain many such hydrophobic secondary metabolites, including terpenoids, furanocoumarins, prenylated flavonoids and some alkaloids^5,24–27^. Although the mechanisms underlying their secretion remain undetermined, it is difficult to determine these mechanisms because only a few types of cells secrete lipids in this manner. For example, secretory cells in glandular trichomes and epithelial cells in oil cavities and resin ducts have been shown to secrete these compounds. Cultured *L. erythrorhizon* cells provide a model system suitable for lipid secretion, because ca. 10% of shikonin is produced as ester derivatives, which are secreted by cells, and plant masses can be easily enlarged in liquid suspension cultures^6^. Our multiple omics study identified 10 genes as being strongly involved in shikonin biosynthesis and 16 as being strongly involved in shikonin secretion^9^. The VIGS methodology described in this study will likely be utilized for functional analysis of these genes to determine the entire shikonin biosynthetic pathway and to understand mechanisms underlying its secretion.

## Materials and methods

### Plant growth conditions

*Lithospermum erythrorhizon* and tobacco plants were grown in soil pots in a room maintained at a temperature of 25 °C and 12 h/12 h day/night conditions. The cultured shoots of *L. erythrorhizon* were grown in a growth chamber set at 25 °C and exposed continuously to light using fluorescent lamps (85 µmol/m^2^ sec on average).

### Vector construction and virus preparation

The trigger fragment of *LePDS1* transcript was amplified from a pool of *L. erythrorhizon* cDNA by RT-PCR, using the primers 5′-GCCCTCGAGCTCCTAAGTGTGTATG-3′ and 5′-CCGGGATCCCACGGACCTCGGAGTC-3′. The amplified fragment was digested with BamHI and XhoI and inserted into the BamHI and XhoI recognition sites of the plasmid pBICAL2^17^. The resulting plasmid and pBICAL1 were introduced into *Agrobacterium* strain GV3101. Colonies on plates of the two strains were mixed in extraction buffer (10 mM MES-KOH, pH 5.7, 10 mM MgCl_2_, 0.15 mM acetosyringone). One of the true leaves of a tobacco plant (*Nicotiana benthamiana*) was covered after sprinkling with carborandom 600 mesh (nacalai tesque, Japan) during the stage of expansion of four leaves. A solution of *Agrobacterium* was placed on the leaf, which was rubbed gently by a hand covered with a rubber glove (rub-inoculation), and the leaf was washed with water to remove the carborandom. After three weeks of growth, the leaves were harvested from the tobacco plants and ground with a mortal and pestle in 1 mL of 100 mM phosphate buffer, pH 7.0, containing 0.3 g of diethyldithiocarbamic acid trihydrate. The lysate was cleared with a 0.45 µm syringe filter, and used to inoculate *L. erythrorhizon*.

### Virus infection of *L. erythrorhizon* by rub-inoculation

The inoculation solution described above was placed onto the true leaves of 21-day-old *L. erythrorhizon* seedlings and rubbed by hand as above. Mock control leaves were inoculated with extraction buffer alone. The inoculated plants were washed with water and grown for 5 weeks to observe the albino phenotype. Total RNA was extracted using Qiagen RNeasy plant mini kits (Qiagen, Germany) and reverse transcribed with ReverTraAce (Toyobo, Osaka, Japan). PCR primers for detecting ALSV containing an integrated trigger fragment were 5′-ACTTCTGATGGTGTCCTCA-3′ and 5′-TAACTCTTGCAAGGTGGTCG-3′.

### Quantification of transcript abundance

Total RNA was extracted and reverse transcribed as above. The *LePDS1* and *LePDS2* transcripts were quantified by BioRad real-time PCR system (Bio-Rad, CA, USA) using the primer pairs 5′-GCCCTCGAGCCTGATGAACTTTC-3′ and 5′-CCGGGATCCCGAACTTCACCACC-3′ and *LeACT7* transcripts were quantified using the primers 5′-TTTTGACTGAGGCACCCC-3′ and 5′-TGACAGGAACTCCCACTAGCT-3′. *LePDS1* and *LePDS2* mRNAs were normalized relative to the levels of *LeACT7* mRNA in the same samples^10^. For amplicon sequencing, *LePDS1* and *LePDS2* cDNAs were PCR amplified using the primers 5′-GTGTACCAGATCGAGTTAC-3′ and 5′-TGACAGGAACTCCCACTAGCT-3′, attached to adaptors and with four nucleotide tag sequences at their 5′ ends. Deep sequencing was performed by Macrogen Japan Corp. (Kyoto Japan) with a MiSeq system. The number of reads identical in *LePDS1* and *LePDS2* sequences were counted in silico with a Perl script.

### Phylogenetic analysis

Amino acid sequences were aligned using a muscle algorithm. A phylogenetic tree was determined using the neighbor-joining method with 1,000 bootstrap replicates and drawn using MEGA7 software^28^. The accession numbers are indicated in Fig. 4C.

### Analysis of shikonin derivatives

Shikonin derivatives in *L. erythrorhizon* were extracted and analyzed quantitatively as described^10^. The extract was analyzed by HPLC as previously reported, with several modifications^29^. Briefly, HPLC was performed on a Prominence system (Shimadzu), which was used with a TSK gel column ODS-80 Tm (4.6 mm × 250 mm, TOSOH); a solvent system consisting of a 7:3 mixture of acetonitrile and water containing 1% acetic acid and 1% triethylamine; a flow rate of 1 ml/min; and an oven temperature of 40 °C; with detection at 520 nm.

## Supplementary information

Supplementary file1 (PDF 11268 kb)

## Abbreviations

qRT-PCR: Quantitative reverse-transcriptase mediated polymerase chain reaction
VIGS: Virus-induced gene silencing
PDS: Phytoene desaturase
ALSV: Apple latent spherical virus
ACT: Actin
UTR: Untranslated region
CMV: Cucumber mosaic virus
