# Supplementary material for: Apple latent spherical virus (ALSV)-induced gene silencing in a medicinal plant, Lithospermum erythrorhizon
Source: Sci Rep. 2020 Aug 11;10:13555. doi: 10.1038/s41598-020-70469-1 (PMC7421898; doi:10.1038/s41598-020-70469-1)
Supplement: Supplementary file 1 — Supplementary file1 (PDF 11268 kb) [file 41598_2020_70469_MOESM1_ESM.pdf]

Supplementary Information

Apple latent spherical virus (ALSV)-induced gene silencing in a medicinal plant, *Lithospermum erythrorhizon*

Yuki Izuishi[1], Natsumi Isaka[1], Hao Li[1], Kohei Nakanishi[1], Joji Kageyama[1], Kazuya Ishikawa[2], Tomoo Shimada[2], Chikara Masuta[3], Nobuyuki Yoshikawa[4], Hiroaki Kusano[1] and Kazufumi Yazaki[1]

1, Laboratory of Plant Gene Expression, Research Institute for Sustainable Humanosphere, Kyoto University, Gokasho, Uji 611-0011, Japan
2, Graduate School of Science, Kyoto University, Sakyo-ku, Kyoto 606-8502, Japan
3, Research Faculty of Agriculture, Hokkaido University, Kita 9 Nishi 9, Kita-ku, Sapporo 060-8589, Japan
4, Agri-Innovation Center, Iwate University, Morioka 3-18-8, Iwate 020-8550, Japan

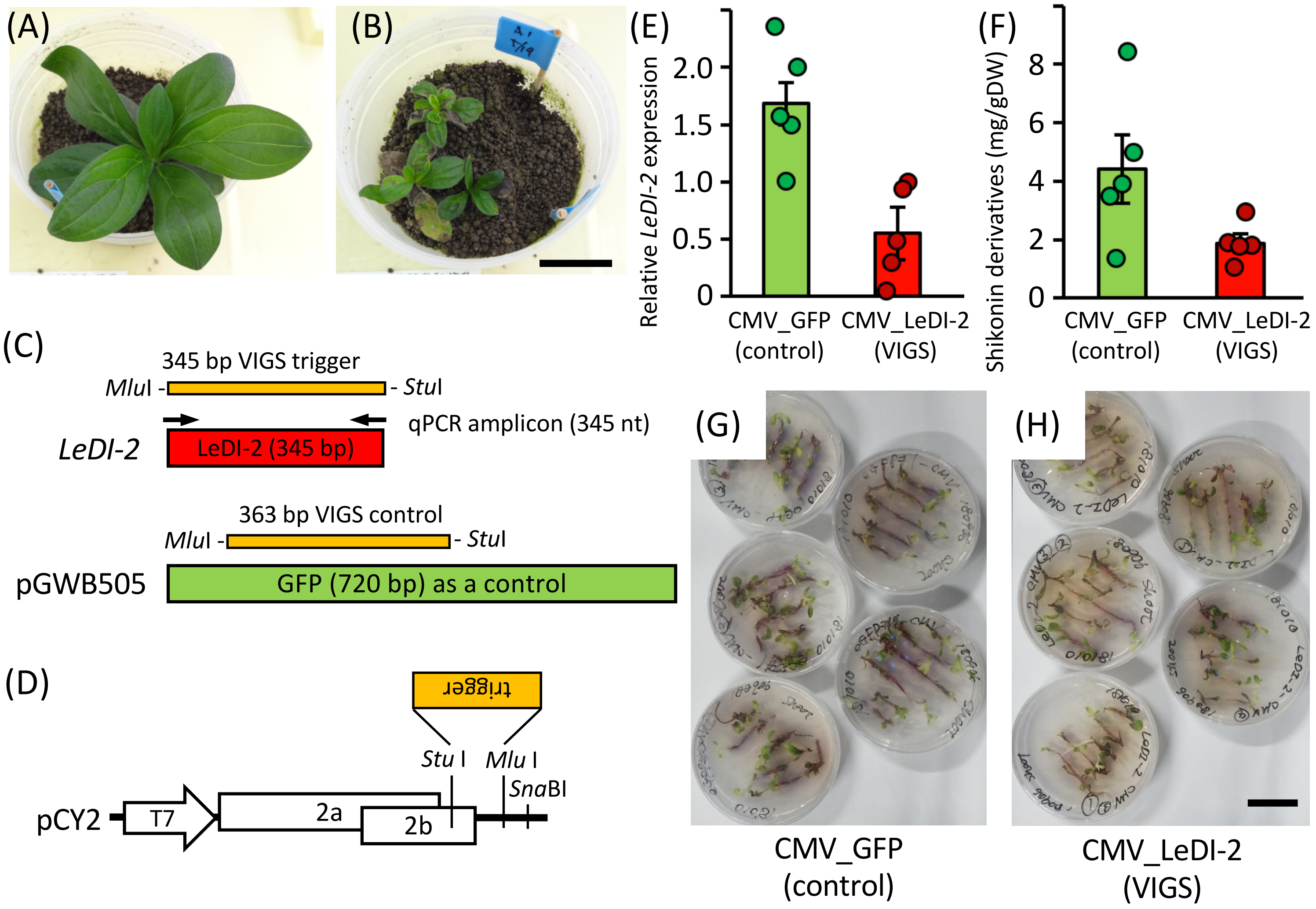

Figure S1. Cucumber mosaic virus (CMV)-mediated gene silencing in *Lithospermum erythrorhizon* tissue-cultured shoots.

(A and B) Photographs of soil-grown plants eight weeks after mock inoculation (A) and virus inoculation (B). Scale bar represents 3 cm. (C) Schematic representations of the target *LeDI-2* gene and the control *GFP* fragment in *L. erythrorhizon*. The PCR primers used to amplify VIGS trigger fragments for the construction of CMVs and to amplify sequences for qPCR were, for *LeDI2*, 5'-GCCACGCGTATGGCTTCAAGAAACTCTATTGCG-3' (forward) and 5'-GCCAGGCCTATTTGGGCAGCTCCATCCAG-3' (reverse), and, for *GFP*, 5'-GCCACGCGTCCTGAAGTTCATCTGCACCA-3' (forward) and 5'-CCGAGGCCTGTTCACCTTGATGCCGTTCT-3' (reverse). (D) Construction of the CMV vector. Amplified trigger fragments were inserted between the StuI and MluI sites, as described (Otagaki et al., 2006). Viral RNAs were transcribed using mMessage mMachine kits (Invitrogen) according to the manufacturer's directions. The three types of viral RNAs were mixed and introduced into *Nicotiana benthamiana* leaves to prepare virus particles by rubinoculation method described in the Materials and Methods section. The inoculation solution prepared from the *N. benthamiana* leaf was inoculated into leaves of cultured shoots of *L. erythrorhizon*. (E) qPCR analysis of *LeDI-2* gene expression and *GFP* fragment as a control, normalized relative to *ACT* gene expression in *L. erythrorhizon* leaves inoculated with CMV_GFP (control) and CMV_LeDI-2 (VIGS). Each bar represents the mean ± SD of five biological replicates. The p value for the difference between the bars was 5.00E-3 by Student's t-test. (F) Contents of shikonin derivatives in stems of *L. erythrorhizon* plants inoculated with CMV_GFP (control) and CMV_LeDI-2 (VIGS). Each bar represents the mean ± SD of five biological replicates. The p value for the difference between the bars was 0.068 by Student's t-test. (G and H) Photographs of cultured *L. erythrorhizon* shoots infected with CMV_GFP (G) andCMV_LeDI-2 (H). The bar represents 3 cm.

Izuishi et al., Figure S1

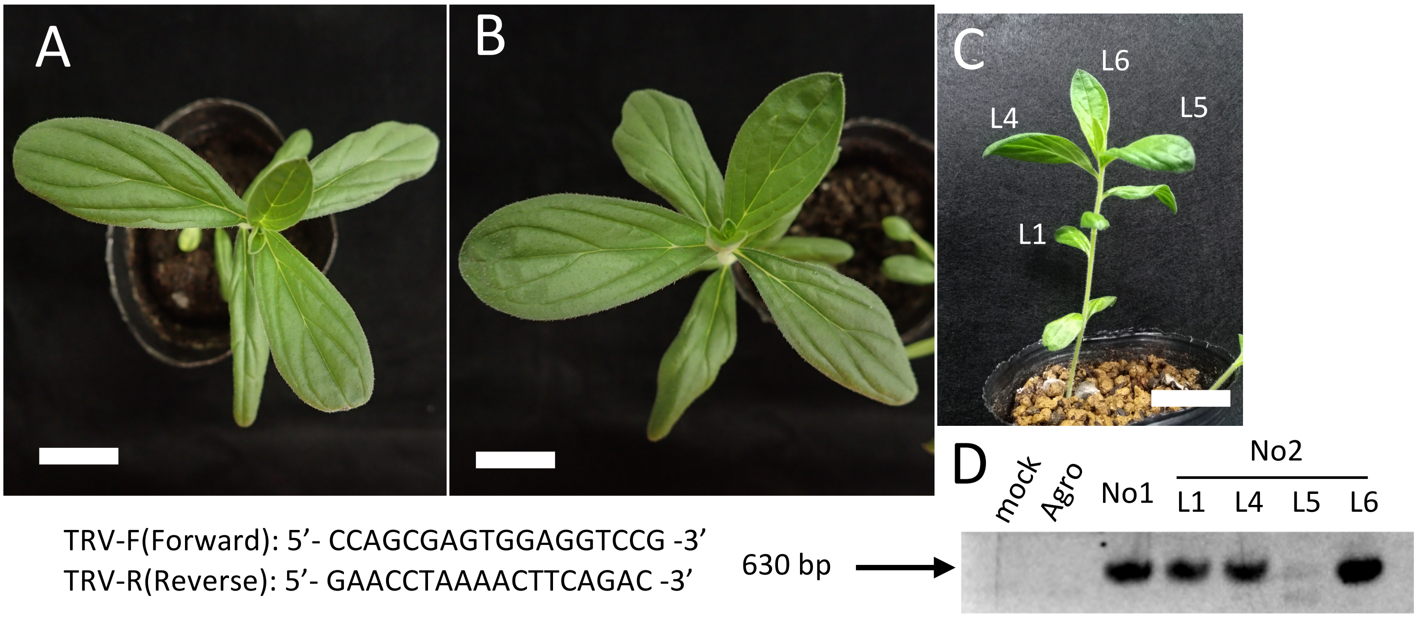

Figure S2. Effect of TRV inoculation on *Lithospermum erythrorhizon* plants.
(A and B) Photographs showing (A) mock-infected and (B) TRV-infected plants. Bar represents 3 cm. (C) Photograph showing leaf levels (L1–L6) of a plant (No. 2 in D) inoculated with TRV. Bar represents 6 cm. (D) Detection of virus in TRV-inoculated plants. Viral RNA was PCR amplified using the primers shown in this figure, and the PCR products were electrophoresed on an agarose gel. Virus was detected in plant No. 1 and in leaves L1, L4 and L6 of plant No. 2, but not in a mock-infected plant or a plant inoculated with *Agrobacterium* solution (Agro).

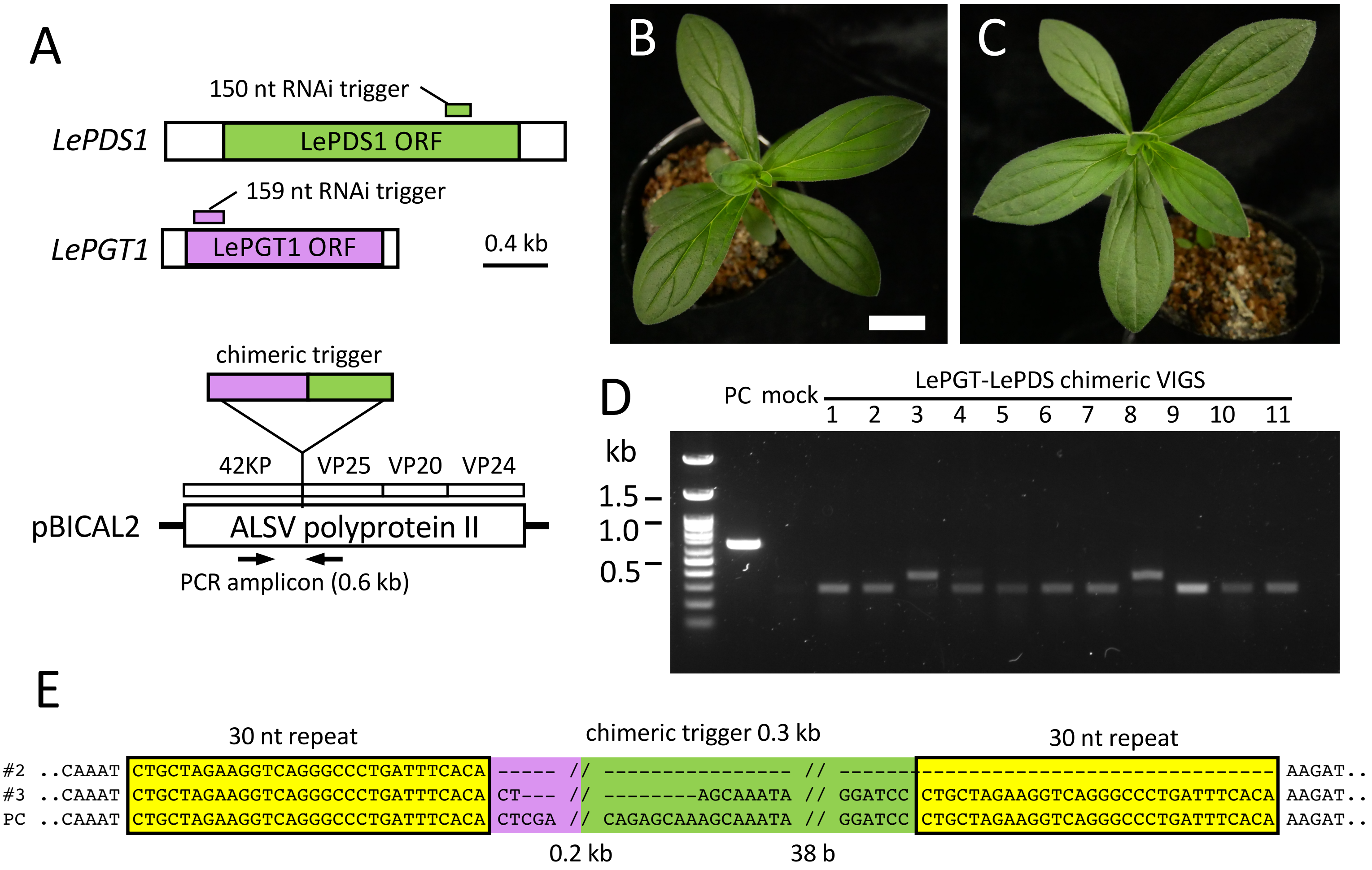

Figure S3. ALSV vector for silencing *LePGT1* in *Lithospermum erythrorhizon*.
(A) Scheme of vector construction. Fragments of *LePGT1* and *LePDS1* cDNA was tandemly inserted into ALSV vector. Primers used for this construction were follows: 5'- GCCCTCGAGCTGATCTTGAAAGTCTGCCT -3' and 5'- GCCGAATTCGTGAGACTGCACCACTAGCA -3' for the *LePGT1* trigger fragment; 5'- GCCGAATTCCCTGCAGAAGAATGGATTTC -3' and 5'- CCGGGATCCCACGGACCTCGGAGTC -3' for the *LePDS1* VIGS trigger fragment. The PCR primers shown are used for virus detection with the same method as applied to *LePDS1* silencing experiment. (B and C) Photographs showing mock-infected (B) and ALSV-infected (plant #3) plants (C). Bar represents 2 cm. (D) Detection of virus-derived RNA in ALSV-treated plants. PC, positive control using the plasmid as template; mock, a mock-infected plant; LePGT-LePDS chimeric VIGS, ALSV-infected plants. PCR products were electrophoresed on an agarose gel. Virus was detected in all plants tested, but shorter in the size. (E) Nucleotide sequence alignment of the PCR fragment from plant #2 and #3 as well as PC. Dashes represents gaps showing the deletion of virus nucleotide sequence. The 30 nt repeat located both side of the trigger sequence.

Izuishi et al., Figure S3

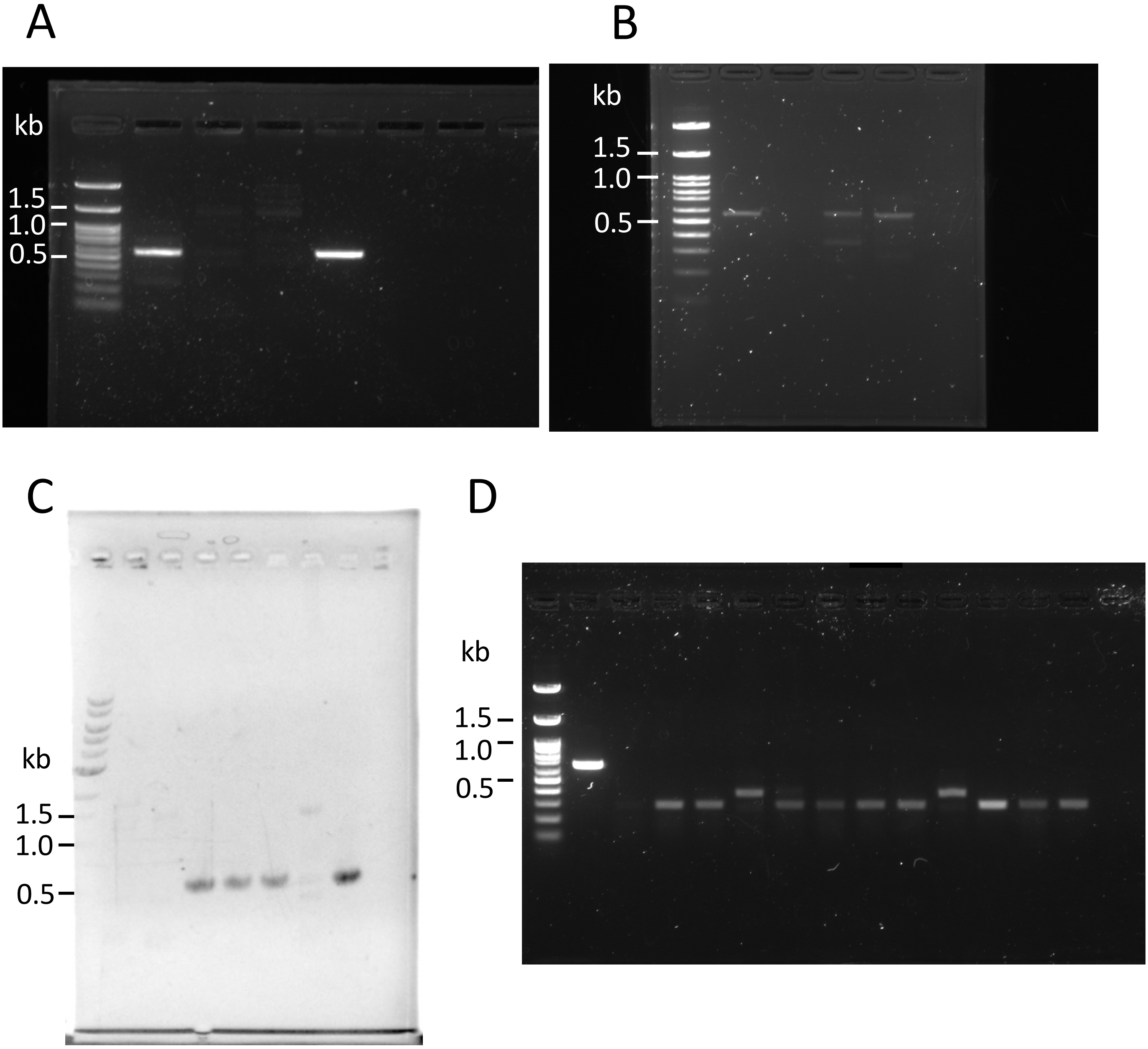

Figure S3. Full-length gels.
(A) Full-length gel of Figure 1E, (B) full-length gel of Figure 3A, and (C) full-length gel of Figure S2D.
